# Deep Eutectic Solvent Synthesis of Perovskite Electrocatalysts for Water Oxidation

**DOI:** 10.1021/acsami.1c24223

**Published:** 2022-05-12

**Authors:** Sangki Hong, Aida M. Díez, Adedoyin N. Adeyemi, Juliana P. S. Sousa, Laura M. Salonen, Oleg I. Lebedev, Yury V. Kolen’ko, Julia V. Zaikina

**Affiliations:** † Department of Chemistry, Iowa State University, Ames, 50011 Iowa, United States; ‡ Nanochemistry Research Group, International Iberian Nanotechnology Laboratory, Braga 4715-330, Portugal; § Laboratoire CRISMAT, UMR 6508, CNRS-ENSICAEN, Caen 14050, France

**Keywords:** complex oxides, electrocatalysis, lattice oxygen evolution reaction, oxygen vacancies, choline chloride

## Abstract

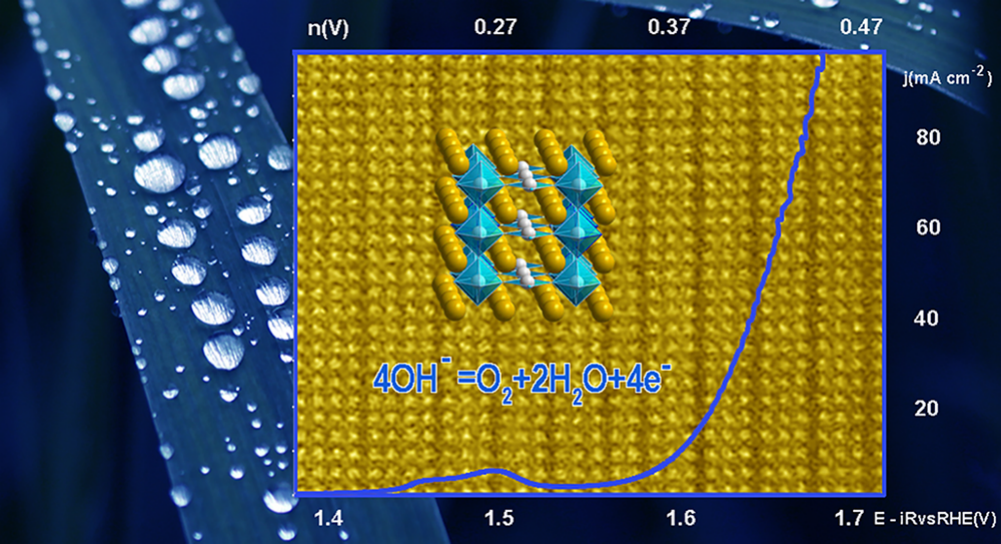

Oxide perovskites have attracted great interest as materials for energy conversion due to their stability and structural tunability. La-based perovskites of 3d-transition metals have demonstrated excellent activities as electrocatalysts in water oxidation. Herein, we report the synthesis route to La-based perovskites using an environmentally friendly deep eutectic solvent (DES) consisting of choline chloride and malonic acid. The DES route affords phase-pure crystalline materials on a gram scale and results in perovskites with high electrocatalytic activity for oxygen evolution reaction. A convenient, fast, and scalable synthesis proceeds via assisted metathesis at a lower temperature as compared to traditional solid-state methods. Among LaCoO_3_, LaMn_0.5_Ni_0.5_O_3_, and LaMnO_3_ perovskites prepared via the DES route, LaCoO_3_ was established to be the best-performing electrocatalyst for water oxidation in alkaline medium at 0.25 mg cm^–2^ mass loading. LaCoO_3_ exhibits current densities of 10, 50, and 100 mA cm^–2^ at respective overpotentials of approximately 390, 430, and 470 mV, respectively, and features a Tafel slope of 55.8 mV dec^–1^. The high activity of LaCoO_3_ as compared to the other prepared perovskites is attributed to the high concentration of oxygen vacancies in the LaCoO_3_ lattice, as observed by high-resolution transmission electron microscopy. An intrinsically high concentration of O vacancies in the LaCoO_3_ synthesized via the DES route is ascribed to the reducing atmosphere attained upon thermal decomposition of the DES components. These findings will contribute to the preparation of highly active perovskites for various energy applications.

## Introduction

Perovskites with a general formula of ABO_3±*x*_ have attracted great interest for energy-related applications due to their structural flexibility and stability. Particularly, La-based perovskites, LaTO_3_ (T = 3d late-transition metal), have demonstrated high electrocatalytic activity for oxygen evolution reaction (OER),^1,2^ which is crucial for rechargeable metal–air batteries as well as water electrolysis. However, overcoming slow kinetics caused by multi-electron transfer remains a great challenge and requires a large overpotential.^3^

To design a cost-effective and high-performance electrocatalyst for OER, substantial efforts have been made to understand the reaction mechanism and ideally to recognize effective activity descriptors.^4−6^ Shao-Horn and co-workers have identified several descriptors relevant to the OER activity of perovskites,^6^ namely the number of d electrons, charge-transfer energy (covalency), and optimal e_g_ orbital occupancy. Additionally, structural factors, such as metal–oxygen–metal bond angles, were found to be relevant. Oxygen vacancies, *V*_O_, have also been shown to play a key role in the OER activity of perovskite oxide catalysts.^7−11^

Deep eutectic solvents (DESs) are emerging green solvents that are inexpensive, non-toxic, and biodegradable,^12^ rendering them interesting media for sustainable synthesis of functional materials. They can be prepared by mixing a hydrogen-bond donor and acceptor (typically quaternary ammonium salt choline chloride) in a desired molar ratio.^13^ Due to the formation of a eutectic, the melting point of the mixture is lowered as compared to its individual components.

We have previously shown that the DES synthesis route facilitates the preparation of complex oxides with mixed-valent states of metal cations,^14,15^ giving access to mixed-valent ternary Zn and Cu vanadates. The reduced metal centers V^4+^ and Cu^+^ were found to modify electronic structures and optical properties of the resultant vanadates. This DES route, giving access to complex oxides with *V*_O_, prompted us to target green synthesis of perovskite electrocatalysts for alkaline OER.

Herein, we present a convenient, scalable, and green DES route to La-based perovskite oxides, LaTO_3_ (T = Mn, Mn/Ni, or Co), comprising dissolution of metal salts and binary metal oxides in environmentally benign solvents followed by heat treatment under air. The resultant materials were tested as electrocatalysts for water oxidation in alkaline medium, and LaCoO_3_ was found to exhibit the best catalytic activity among the prepared perovskites. High-resolution transmission electron microscopy (HRTEM) studies were carried out to gain insights into the structures, and a plausible reaction mechanism is discussed.

## Results and Discussion

We developed a green DES synthesis route for LaCoO_3_, LaMn_0.5_Ni_0.5_O_3_, and LaMnO_3_ perovskites due to the expected high electrocatalytic activity of these materials in alkaline OER.^5,16,17^ The DES of choice was a eutectic mixture of malonic acid (mp 135–137 °C) as the hydrogen-bond donor and choline chloride (mp 302 °C) as the hydrogen-bond acceptor mixed in a 1:1 molar ratio. The resulting eutectic mixture has a melting point of 10 °C. The binary precursors manganese oxide Mn_2_O_3_ and metal salts (LaCl_3_, NiCl_2_, and CoCl_2_) were dissolved in this medium at 70 °C. The produced solutions were then calcined at moderate temperatures of 200–400 °C in an open crucible under air to remove NH_3(g)_ and HCl_(g)_ side products stemming from the decomposition of the DES resulting in semi-amorphous solids. Upon further calcination at 900–1000 °C, the leftover hydrocarbon was removed via combustion, resulting in the crystallization of the target perovskites.

**Figure 1:**
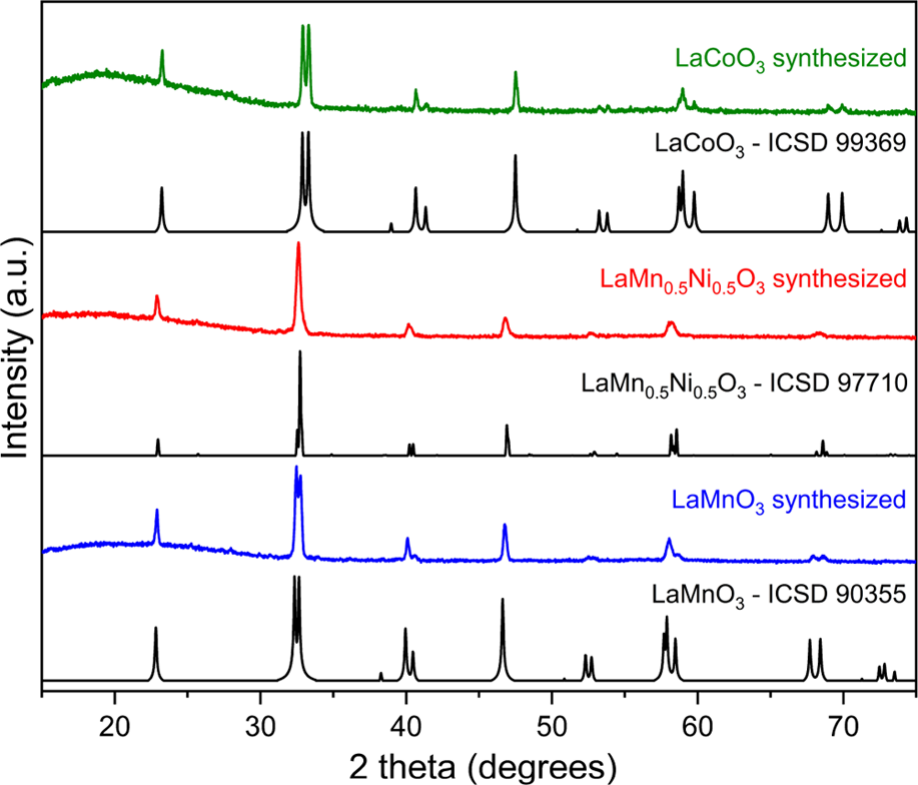
Collected PXRD and calculated reference patterns of DES-derived perovskites.

As determined by powder X-ray diffraction (PXRD), our DES route provides phase-pure perovskites LaCoO_3_, LaMn_0.5_Ni_0.5_O_3_, and LaMnO_3_ (Figure 1). Structural parameters from Rietveld refinement are summarized in Figure S1 and Table S1.

The PXRD patterns of LaTO_3_ (T = Mn, Co) indicate a rhombohedral crystal structure with the space group *R*3̅c. TO_6_ octahedra are slightly tilted, namely rotated about the threefold rotation axis as compared to a cubic perovskite (Figure S2). The tilt angles (4.697° for Mn and^18^ 5.3565° for Co^19^) are small due to the large ionic radius of 12-coordinated trivalent La.^20^ A significantly larger unit cell volume of LaMnO_3_ cf. LaCoO_3_ arises from the greater ionic radius of high-spin Mn^3+^ cf. Co^3+^ in the octahedral environment (Table S1).

The PXRD pattern of LaMn_0.5_Ni_0.5_O_3_ is indexed in a monoclinic unit cell (*P*2_1_/*c*, Figure S3a,b), although the possibility of a rhombohedral polymorph (*R*3̅, Figure S3c,d) present as a minor phase cannot be ruled out due to the similarity of the PXRD patterns. Moreover, the structural transition from a low-temperature monoclinic phase to a high-temperature rhombohedral phase occurs at room temperature according to Blasco and co-workers.^21^ Notably, there are two distinct T–O (T = Mn, Ni) distances: 1.902 Å, which is typical for Mn^4+^ in metal oxides and 2.025 Å, which is characteristic of Ni^2+^.^21^ As a result, the different octahedral environments introduce significant distortion in the perovskite structure and create two crystallographically independent B-sites. Neutron diffraction studies have revealed that there is a site preference for Mn and Ni cations in both polymorphs, classifying LaMn_0.5_Ni_0.5_O_3_ as a double perovskite with a nominal formula La_2_MnNiO_6_.^21,22^

As compared to traditional solid-state synthesis methods,^23,24^ the DES methodology reported here provides rapid access to perovskite materials without the need of intermediate regrinding/reheating efforts or reaction temperatures >1000 °C. The DES protocol not only facilitates homogeneous mixing of metal cations in the solution but also favors the formation of reactive intermediates. Specifically, a labile ternary intermediate, LaOCl, was found to form at ≥400 °C (Figure S4), which further reacts with binary metal oxides (Mn_3_O_4_, NiO, or Co_3_O_4_) at 900 °C or 1000 °C to form the respective perovskites. According to the literature, these findings suggest that our synthesis follows an assisted metathesis reaction pathway. In particular, Todd and co-workers reported synthesis of YMnO_3_ via assisted metathesis.^25,26^ The reaction between Mn_2_O_3_, YCl_3_·6H_2_O, and Li_2_CO_3_ can proceed according to
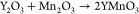
1in which binary oxides react at 600 °C, or according to
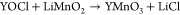
2which is an example of metathesis reaction.^25,26^ The latter reaction is faster and stabilizes metastable orthorhombic YMnO_3_ at a lower temperature of 500 °C because of reactive ternary intermediates yttrium oxochloride YOCl and lithium manganese oxide LiMnO_2_.^25,26^ Considering that our DES route does not require multiple grinding steps or reheating at elevated temperatures, we conclusively prove that LaOCl is a kinetically labile intermediate in the DES synthesis of La-based perovskite oxides (Figure S4), similar to the assisted metathesis reaction pathway in the reported synthesis of YMnO_3_.

Next, we investigated the electrocatalytic activity of the newly synthesized perovskites in alkaline OER. To facilitate the preparation of the electrocatalytic inks and to increase the surface area for improved OER activity, the materials were ball-milled into fine powders. Notably, the ball-milling process does not result in structural changes in the materials but slightly decreases the average crystallite size (Table S2) and induces strain. The ball-milled perovskites were then formulated as inks in ethanol-containing conductive Nafion ionomer as a binder,^27^ and the ink was deposited onto the Ni foam current collector. More details on the anode fabrication and electrochemical testing can be found in the Supporting Information. Experimentally, the perovskites were found to demonstrate the best OER properties at 0.25 mg cm^–2^ mass loading (Figure S5). In addition, the perovskites required prolonged activation via cyclic voltammetry (CV) for about 700 cycles to achieve steady-state conditions (Figure S6), after which the materials exhibit constant OER performance.

**Figure 2:**
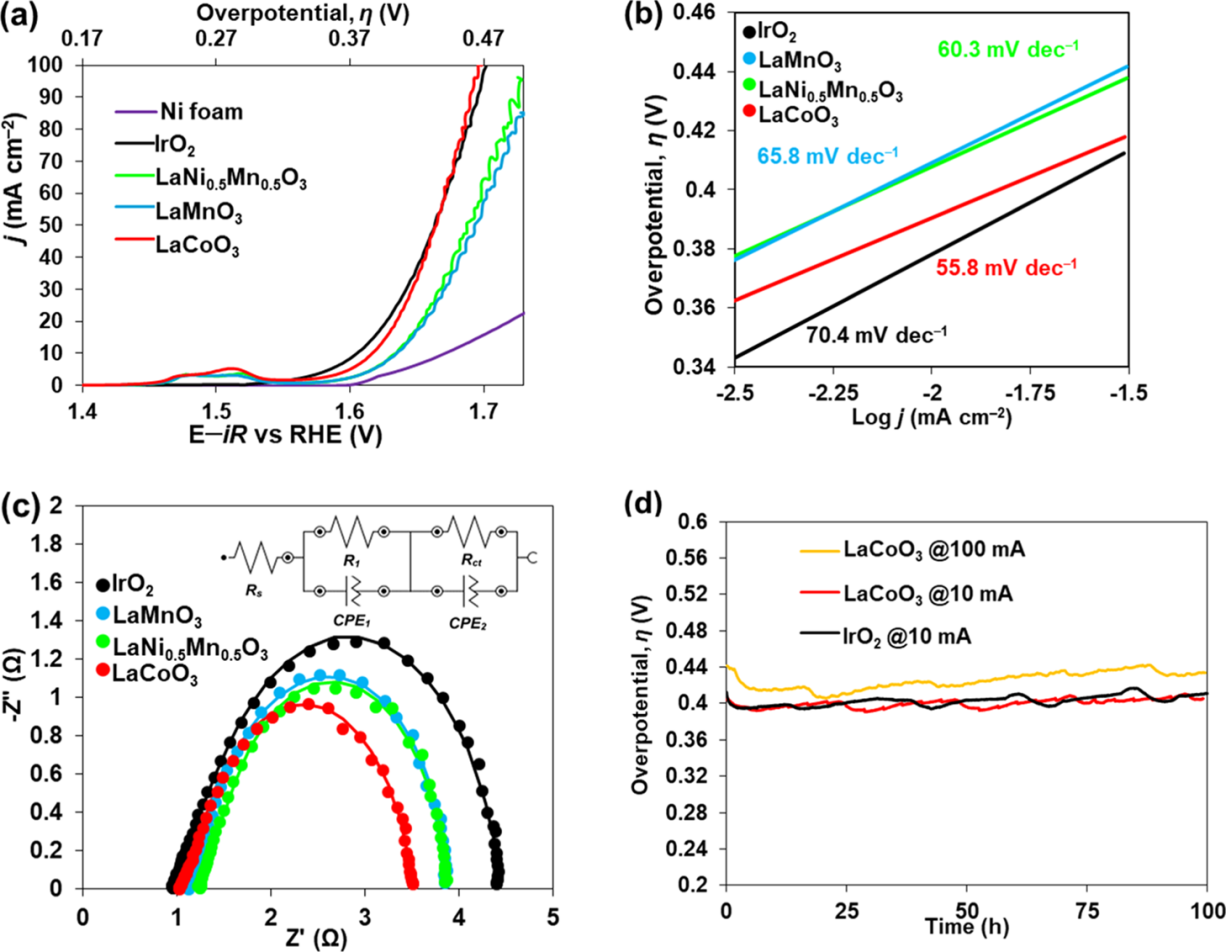
Anodic polarization curves (a), Tafel (b), Nyquist (c), and chronopotentiometric (d) plots for Ni foam-supported perovskites and reference IrO_2_, all recorded in 1 M NaOH at room temperature with a mass loading of 0.25 mg cm^–2^. The inset in (c) shows an equivalent electrical circuit model used to fit the Nyquist plots.

Figure 2 and Table S3 summarize the electrochemical water oxidation properties in 1.0 M NaOH of DES-synthesized, ball-milled LaCoO_3_, LaMn_0.5_Ni_0.5_O_3_, and LaMnO_3_ electrocatalysts supported on Ni foam, in comparison with the standard reference IrO_2_ electrocatalyst supported on Ni foam as well as pure Ni foam. High electrocatalytic performance was observed for all prepared electrocatalysts (Figure 2a). LaCoO_3_ affords the most favorable apparent OER properties based on the geometric area, with current densities *j* of 10, 50, and 100 mA cm^–2^ reached at overpotentials η of around 390 (η_10_), 430 (η_50_), and 470 (η_100_) mV, respectively (Table S3). Notably, both Mn-containing perovskites, LaMnO_3_ and LaMn_0.5_Ni_0.5_O_3_, have comparable OER properties, while exhibiting higher OER overpotentials as compared to the best-performing LaCoO_3_ electrocatalyst (Figure 2a, Table S3). The shallow Tafel slope *b* = 55.8 mV dec^–1^ was estimated for LaCoO_3_, while LaMnO_3_ and LaMn_0.5_Ni_0.5_O_3_ demonstrate slightly higher values of *b* = 65.8 mV dec^–1^ and *b* = 60.3 mV dec^–1^, respectively (Figure 2b, Table S3).

Notably, significant capacitive currents were observed for perovskites between approximately 1.40 and 1.55 V in Figure 2a, which could be indicative of a variation in the electrochemically active surface area (ECSA) of the electrocatalysts. To investigate how the intrinsic OER activities of LaCoO_3_, LaMn_0.5_Ni_0.5_O_3_, and LaMnO_3_ materials compare, the activity data in Figure 2a were normalized to the measured double-layer capacitance *C*_dl_ (Figure S7, Table S3). Assuming that all the perovskites have a similar specific capacitance, *C*_p_, and neglecting the contribution of cation intercalation into the material, ECSA can be inferred to be proportional to *C*_dl_. From the resultant normalized data shown in Figure S8, one can conclude that the intrinsic activity of LaCoO_3_ (per unit ECSA) is higher than that of LaMn_0.5_Ni_0.5_O_3_ and LaMnO_3_, and thus, its better performance is not simply related to its higher ECSA.

To gain insights into the OER kinetics assuming aqueous electrochemical assembly,^28^ electrochemical impedance spectroscopy experiments were carried out on a stable electrocatalytic system at ≈η_10_, as illustrated by the Nyquist plots (Figure 2c). Experimental data were fitted using an equivalent circuit model (Figure 2c, inset) accounting for a resistor, which is in series with parallel combination of a resistor and a constant phase element. Specifically, the resistor *R*_s_ is the Ohmic resistance from the electrolyte and all contacts. The time constant *R*_1_–CPE1 is associated with the interfacial resistance from electron transport between the electrocatalyst and Ni foam current collector, while the time constant *R*_ct_–CPE2 is the charge-transfer resistance (*R*_ct_) at the catalyst–electrolyte interface.^29^ Importantly, shallow *R*_ct_ values typically reflect faster charge-transfer kinetics.^29^ The analysis of the obtained semi-circles (Figure 2c) as well as derived parameters (Table S3) reveals fastest charge-transfer kinetics over the anode/electrolyte interface in the case of Ni foam-supported LaCoO_3_, as reflected by its smallest *R*_ct_ = 2.1692 Ω, compared to Ni foam-supported LaMn_0.5_Ni_0.5_O_3_ (*R*_ct_ = 2.6762 Ω) and LaMnO_3_ (*R*_ct_ = 2.7260 Ω).

Interestingly, we observed that the CV curves of the perovskites feature pre-oxidation peaks at potentials lower than their respective OER onsets (Figures 2a, S5, and S6). The trend of the pre-oxidation peak potential, LaCoO_3_ (1.51 V) > LaMnO_3_ ≈ LaMn_0.5_Ni_0.5_O_3_ (1.48 V), agrees well with that of charge-transfer resistance *R*_ct_, LaCoO_3_ < LaMnO_3_ ≈ LaMn_0.5_Ni_0.5_O_3_ (Table S3). These observed pre-oxidation peaks are characteristic of Co^3+^/Co^4+^ and Mn^3+^/Mn^4+^ redox couples in the respective perovskites, indicating oxidation of the electrocatalysts during alkaline OER.

Having established that LaCoO_3_ demonstrates excellent OER properties, we further investigated if this electrocatalyst can generate a stable current during alkaline OER. To this end, the anode was subjected to continuous stability testing by means of chronopotentiometry, where LaCoO_3_ showed excellent long-term stability in 1 M NaOH electrolyte during the tested 100 h, affording reasonably steady current density *j* of 10 and 100 mA cm^–2^ at overpotentials η of only ≈390 and 450 mV, respectively (Figure 2d).

The activity and stability of the synthesized perovskite electrocatalysts were also compared to the standard platinum group metal reference IrO_2_ electrocatalyst at the same mass loading of 0.25 mg cm^–2^ (Figure 2). Despite possible differences in the surface area between LaCoO_3_ and IrO_2_, the perovskite can be deduced to offer at least comparable OER properties to the significantly more expensive and less abundant reference electrocatalyst. Overall, the low OER overpotential, shallow Tafel slope, fast kinetics, and stable long-term performance of DES-synthesized LaCoO_3_ (Figure 2, Table S3) highlight the potential of this material in electrolysis applications.

To rationalize the observed excellent activity and stability of our LaCoO_3_ in alkaline water oxidation, we carried out detailed electron microscopy investigations of the material before and after electrocatalytic testing. Figure S9 shows a representative *z*-contrast high-angle annular dark-field scanning transmission electron microscopy (HAADF–STEM) image of the sample after ball milling but before OER, together with the corresponding energy-dispersive X-ray spectroscopy mapping in the STEM mode (STEM–EDX). These images indicate that La, Co, and O elements are uniformly distributed across the sub-μm-sized LaCoO_3_ particles. Notably, neither N nor Cl elements are present in the as-synthesized LaCoO_3_, confirming that DES constituents are eliminated during combustion reaction. This contrasts with the recent report about N doping into O sites in LaCoO_3_ prepared by annealing of the pristine oxide powder in the atmosphere of ammonia gas.^30^

**Figure 3:**
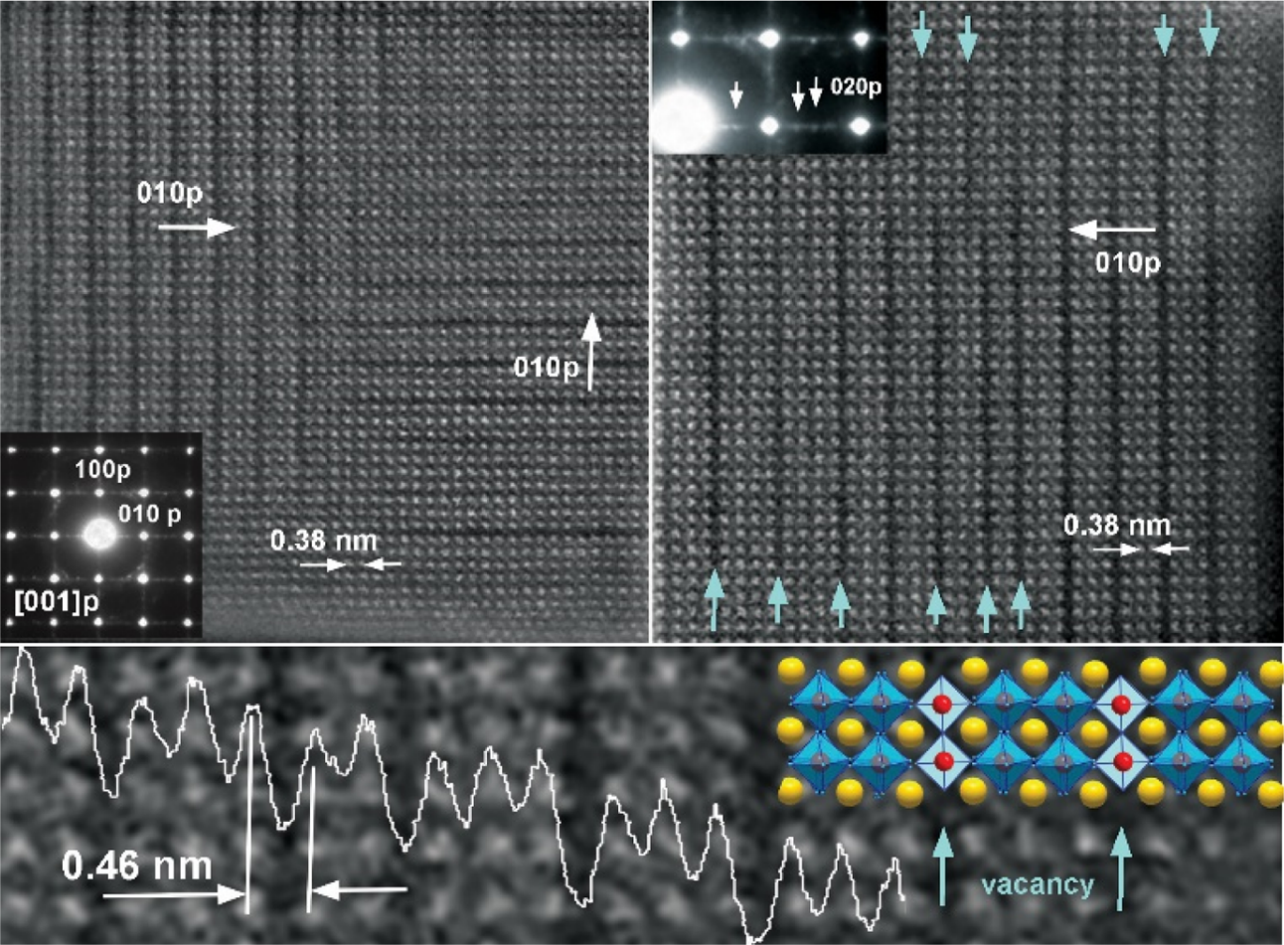
Top panel: representative [010] HAADF–STEM images of LaCoO_3_ after ball milling together with the corresponding SAED patterns (insets) evidencing dark stripes of *V*_O_. White arrows in the magnified selection of the SAED pattern (right top panel inset) indicate superstructure spots. Bottom panel: magnified HRTEM image with an overlaid structural model and intensity line scan profile showing periodic change of La–La distances from typical 0.38 nm to expanded 0.46 nm in *V*_O_ stripes.

Interestingly, high-resolution HAADF–STEM and selected area electron diffraction (SAED) analyses of LaCoO_3_ before OER evidence the existence of a large number of oxygen vacancies, *V*_O_, in the sample (Figure 3) clearly manifested in the HAADF–STEM images by the presence of dark stripes in the Co–O layers. *V*_O_ are also highlighted by the appearance of weak superstructure spots in the respective SAED patterns, which are attributed to the ordering of the dark stripes. The measurement of the interlayer distances revealed the expansion of the La–La distance from a typical 0.38 to 0.46 nm in the *V*_O_ stripe. Both the appearance of dark contrast Co–O layers and the increase in La–La distances can be attributed to *V*_O_ layers, as has been previously observed in perovskite structures.^31−33^ Such layers of *V*_O_ change Co coordination from CoO_6_ octahedral to CoO_5_ square pyramidal. The formation of *V*_O_ in the DES-synthesized perovskites is consistent with our previous studies,^14,15^ where we used a DES route to prepare mixed-valent ternary Zn and Cu vanadates (*M*_2_V_2_O_7–*m*_ and *M*V_2_O_6–*n*_, *M* = Zn or Cu) and found annealing the reaction mixture to intrinsically introduce *V*_O_.

**Figure 4:**
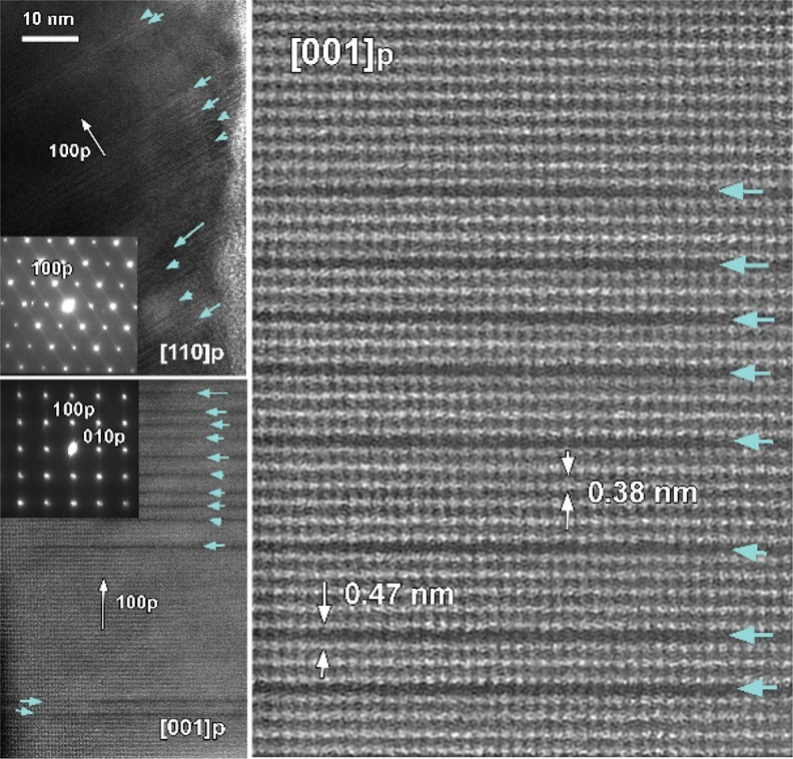
Representative low-magnification [110] TEM and [001] HAADF–STEM (left panels) as well as high-resolution [001] HAADF–STEM (right panel) images together with the corresponding SAED patterns (insets) for LaCoO_3_ after alkaline OER stability testing at a constant current density of 10 mA cm^–2^ for 100 h (Figure 2d), highlighting that *V*_O_ and their ordering have been preserved in the structure.

Remarkably, to the best of our knowledge, there are no studies reporting the formation of *V*_O_ in bulk LaCoO_3_ prepared without doping of metal sites or any post-treatment under reducing conditions, and so far, such examples have only been reported in LaCoO_3_ films.^32,34^ Even annealing of pristine LaCoO_3_ in the reducing atmosphere of ammonium gas does not induce the formation of *V*_O_, with HAADF–STEM of N-doped LaCoO_3_ indicating a regular perovskite structure.^30^ Most notably, transmission electron microscopy (TEM), HAADF–STEM, and SAED investigations of our LaCoO_3_ electrocatalyst after stability testing at a constant current density of 10 mA cm^–2^ for 100 h (Figure 2d) clearly indicate that the *V*_O_ are preserved in the structure of spent LaCoO_3_ (Figure 4).

**Figure 5:**
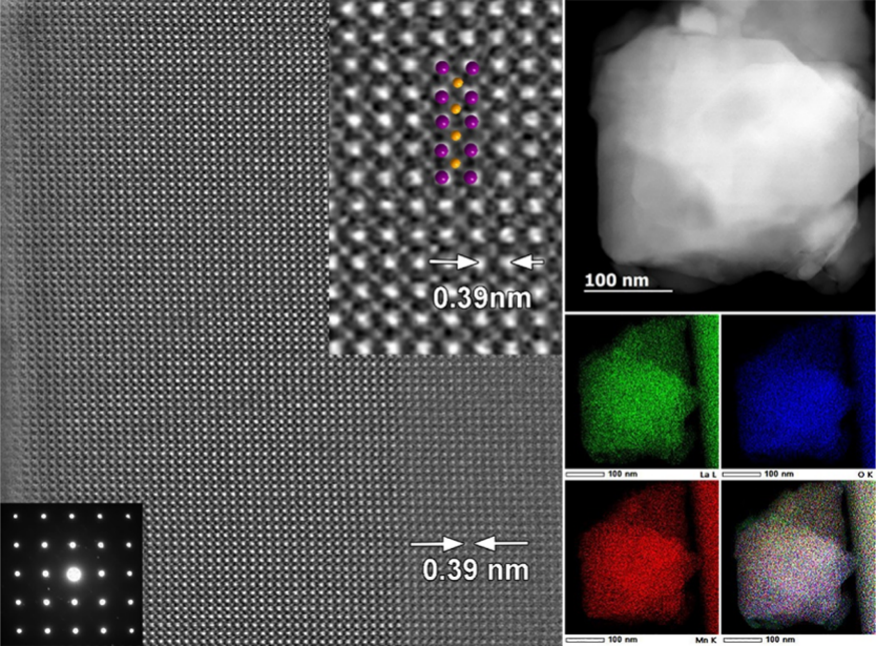
Left panel: Representative HAADF–STEM images of LaMnO_3_ after ball milling, together with the corresponding SAED pattern (insets), evidencing the absence of *V*_O_ in the material. White arrows indicate the typical La–La distances of 0.39 nm. Right panel: HAADF–STEM image with simultaneously collected STEM–EDX mappings of La, O, Mn, and their mixture, indicating a homogeneous distribution of all the elements within the sample.

Interestingly, the high-resolution HAADF–STEM and SAED study of the LaMnO_3_ electrocatalyst with the lower electrocatalytic activity for OER as compared to LaCoO_3_ showed no evidence of *V*_O_ (Figure 5). Typical La–La distances of 0.39 nm were observed in the structure. We hypothesize that such a difference in the *V*_O_ formation between LaCoO_3_ and LaMnO_3_ can be attributed to the higher reduction potential of Co^3+^/Co as compared to Mn^3+^/Mn, affording greater stability of the +3 oxidation state for Mn in LaTO_3_ as compared to Co. Thus, partial reduction under the conditions of our DES route is more likely to occur in the case of LaCoO_3_. Similarly, no evidence of *V*_O_ was found in the case of LaMn_0.5_Ni_0.5_O_3_ (data not shown).

For the LaCoO_3_ catalyst reported herein, a plausible electrocatalytic mode of action is consistent with lattice OER (LOER).^4,5^ Although OER over perovskites or other oxides can proceed via a conventional four-consecutive proton-coupled electron transfer mechanism with metal cations as the catalytically active sites,^35^ examples of alternative or simultaneously occurring LOER mechanisms are increasingly reported in the literature.^36^ In LOER, the oxidation of the lattice oxygen of the electrocatalyst is accompanied by continuous dissolution–redeposition of metal cations, which leads to O_2_ evolution.^37^ The resultant *V*_O_, formed by the evolution of the lattice O, are then compensated by OH^–^ from the alkaline electrolyte,^38^ affording stability to the electrocatalyst and preventing collapse due to depletion in structural O. Furthermore, the LOER mechanism has been demonstrated to be closely associated with the presence of *V*_O_ in perovskites. For example, in La_1–*x*_Sr_*x*_CoO_3−δ_, the existence of *V*_O_ accelerates the dissolution of La/Sr via the LOER mechanism, thus facilitating the in situ reconstruction of the perovskite surface into a highly active CoO(OH) shell.^39^ Accordingly, *V*_O_ have proven to be one of the main factors governing the activity of perovskites in alkaline OER,^40^ somewhat similar to the field of solid oxide fuel cells.^41^

In our study, dissolution of the surface La^3+^ occurs upon OER, resulting in the segregation of a La- and O-containing phase next to the well-isolated LaCoO_3_ particles as indicated by STEM–EDX analysis (Figure S10) of LaCoO_3_ after OER stability testing for 100 h (Figure 2d). This is most likely La(OH)_3_, formed by the precipitation of the as-leached La^3+^ in NaOH electrolyte solution under anodic oxidation conditions.^42^ The surface of LaCoO_3_ could then be reconstructed in situ into a tiny Co-rich shell, perhaps CoO(OH).^39^ In our previous studies, we clearly confirmed the in situ formation of active and stable shells of Fe, Co, or Ni oxide/oxyhydroxide nanoparticles during OER over various phosphide and boride pre-catalysts.^43−46^ In sharp contrast, the formation of such a Co oxide/oxyhydroxide shell was not observed by TEM on the surface of our spent LaCoO_3_ (Figure 4). As such, the process of the formation of a Co-rich shell over our LaCoO_3_ may be rather dynamic owing to the continuous dissolution–redeposition of Co.^39^ Furthermore, the thus-formed Co-rich shell is suggested to deliver O_2_ through anodic oxidation of lattice O in LaCoO_3_. Most notably, the presence of a large number of *V*_O_ in LaCoO_3_ is proposed to enhance its OER activity via high oxygen diffusivity,^7^ thus affording efficient refilling of the evolved lattice oxygen.^40,47^ The fact that *V*_O_ remain after 100 h of alkaline OER over our LaCoO_3_ (Figure 4) indicates that there is a dynamic equilibrium state between electrolyte–surface–bulk during OER.^39,40,48^ Importantly, inductively coupled plasma optical emission spectroscopy chemical analysis of the electrolyte after alkaline OER testing did not reveal any traces of La, Ni, Co, or Mn but merely the presence of 0.022 ppm of Pt admixture, which, most likely, stems from leaching of the Pt wire counter electrode.

**Figure 6:**
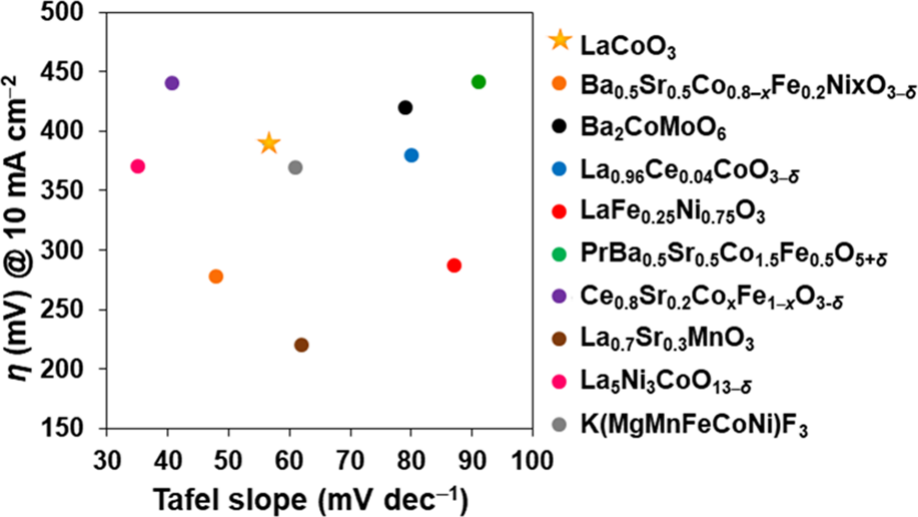
Comparison between the Tafel slopes as well as overpotentials required for driving current density of 10 mA cm^–2^ for our best-performing electrocatalyst, LaCoO_3_, and reported state-of-the-art perovskite electrocatalysts for alkaline water oxidation.^8,9,49−54^

Perovskites featuring oxygen vacancies have been shown to be highly active electrocatalysts for alkaline water oxidation. For example, plasma treatment was used to introduce *V*_O_ in PrBa_0.5_Sr_0.5_Co_1.5_Fe_0.5_O_5+δ_, resulting in highly active catalyst material with *b* = 94 mV dec^–1^.^8^ In another study, the four-layered perovskite La_5_Ni_3_CoO_13−δ_, synthesized through glycine nitrate combustion, was found to be enriched with oxygen defects and showed exceptional electrocatalytic OER activity (*b* = 35 mV dec^–1^).^49^ Ce-doping of LaCoO_3_ was found to significantly increase the concentration of *V*_O_ as compared to pristine LaCoO_3_, with La_0.96_Ce_0.04_CoO_3−δ_ synthesized using a microwave/ultrasound-assisted hydrothermal route (*b* = 80 mV dec^–1^), outperforming pure LaCoO_3_ (*b* = 124 mV dec^–1^) in alkaline OER.^9^ The herein reported DES synthesis route provides a straightforward approach to introduce *V*_O_ in LaCoO_3_, placing the material with η_10_ = 390 mV and *b* = 55.8 mV dec^–1^ among the best of the state-of-the-art perovskite electrocatalysts for alkaline water oxidation (Figure 6).

## Conclusions

In summary, we have prepared a series of La-containing perovskites LaTO_3_ (*T* = Mn, Mn/Ni, or Co) using an environmentally friendly DES synthesis route. DES synthesis enables uniform mixing of metal precursors and the formation of labile intermediates, while the unique reaction environment achieved upon DES calcination facilitates formation of oxygen vacancies in the LaCoO_3_ structure. LaCoO_3_ was found to be a stable electrocatalyst with OER activity among the best reported for perovskites. The high activity was attributed to the presence of a large number of structural oxygen vacancies as evidenced by electron microscopy. Further studies that address a deeper understanding of the effect of oxygen vacancies on OER over DES-derived perovskites, as well as the optimization of their OER properties via nanostructuring, will be the subjects of our future research efforts.

## Experimental Section

### Catalyst Synthesis

The DES was prepared by mixing malonic acid and choline chloride in a 1:1 molar ratio. The mixture was heated in a beaker at 70 °C until it became liquid. Metal precursors were added to the solvent and dissolved under vigorous stirring at 70 °C (0.7158 g of LaCl_3_ and 0.2304 g Mn_2_O_3_ in 87 g of the solvent for LaMnO_3_ | 0.5368 g of LaCl_3_, 0.0865 g Mn_2_O_3_, and 0.1418 g NiCl_2_ in 72.5 g of the solvent for LaMn_0.5_Ni_0.5_O_3_ | 0.6135 g of LaCl_3_ and 0.3248 g CoCl_2_ in 80.0 g of the solvent for LaCoO_3_). Afterward, the metal-precursor-containing solution was transferred to a porcelain crucible for calcination (7 g of solution in a 30 mL crucible). The heat treatment was performed in a box-type muffle furnace for all the samples. For all heat treatments, a heating rate of 10 °C min^–1^ was used.

For the synthesis of LaMnO_3_ and LaMn_0.5_Ni_0.5_O_3_, the precursor solution was heated at 230 °C for 2 h and then calcined at 900 °C for LaMnO_3_ or 1000 °C for LaMn_0.5_Ni_0.5_O_3_ in the span of 8.5 h. LaCoO_3_ was prepared by heating the precursor solution at 300 °C for 2 h, followed by calcination at 900 °C for 8.5 h. Calcination was conducted in an open crucible under the air. Afterward, the samples were cooled to room temperature by switching off the furnace.

## Supporting Information Available

Additional XRD patterns, figures of crystal structures, additional electrocatalytic data, HAADF–STEM images, STEM–EDX elemental maps, tables with structural data, calculated crystallite sizes, and electrocatalytic alkaline OER properties (PDF)

## References

[ref1] Zhu H.; Zhang P.; Dai S. Recent Advances of Lanthanum-Based Perovskite Oxides for Catalysis. ACS Catal. 2015, 5, 6370–6385. 10.1021/acscatal.5b01667.

[ref2] Rana M.; Mondal S.; Sahoo L.; Chatterjee K.; Karthik P. E.; Gautam U. K. Emerging Materials in Heterogeneous Electrocatalysis Involving Oxygen for Energy Harvesting. ACS Appl. Mater. Interfaces 2018, 10, 33737–33767. 10.1021/acsami.8b09024. 30222309

[ref3] Fabbri E.; Schmidt T. J. Oxygen Evolution Reaction—The Enigma in Water Electrolysis. ACS Catal. 2018, 8, 9765–9774. 10.1021/acscatal.8b02712.

[ref4] Beall C. E.; Fabbri E.; Schmidt T. J. Perovskite Oxide Based Electrodes for the Oxygen Reduction and Evolution Reactions: The Underlying Mechanism. ACS Catal. 2021, 11, 3094–3114. 10.1021/acscatal.0c04473.

[ref5] Zhao J.-W.; Shi Z.-X.; Li C.-F.; Ren Q.; Li G.-R. Regulation of Perovskite Surface Stability on the Electrocatalysis of Oxygen Evolution Reaction. ACS Mater. Lett. 2021, 3, 721–737. 10.1021/acsmaterialslett.1c00018.

[ref6] Hong W. T.; Welsch R. E.; Shao-Horn Y. Descriptors of Oxygen-Evolution Activity for Oxides: A Statistical Evaluation. J. Phys. Chem. C 2016, 120, 78–86. 10.1021/acs.jpcc.5b10071.

[ref7] Mefford J. T.; Rong X.; Abakumov A. M.; Hardin W. G.; Dai S.; Kolpak A. M.; Johnston K. P.; Stevenson K. J. Water Electrolysis on La_1–*x*_Sr_*x*_CoO_3−δ_ Perovskite Electrocatalysts. Nat. Commun. 2016, 7, 11053 10.1038/ncomms11053. 27006166 PMC4814573

[ref8] Zhu Y.; Zhong X.; Jin S.; Chen H.; He Z.; Liu Q.; Chen Y. Oxygen Defect Engineering in Double Perovskite Oxides for Effective Water Oxidation. J. Mater. Chem. A 2020, 8, 10957–10965. 10.1039/d0ta04362a.

[ref9] Ji D.; Liu C.; Yao Y.; Luo L.; Wang W.; Chen Z. Cerium Substitution in LaCoO_3_ Perovskite Oxide as Bifunctional Electrocatalysts for Hydrogen and Oxygen Evolution Reactions. Nanoscale 2021, 13, 9952–9959. 10.1039/d1nr00069a. 34076006

[ref10] Li X.; Zhao H.; Liang J.; Luo Y.; Chen G.; Shi X.; Lu S.; Gao S.; Hu J.; Liu Q.; Sun X. A-Site Perovskite Oxides: An Emerging Functional Material for Electrocatalysis and Photocatalysis. J. Mater. Chem. A 2021, 9, 6650–6670. 10.1039/d0ta09756j.

[ref11] Petrie J. R.; Jeen H.; Barron S. C.; Meyer T. L.; Lee H. N. Enhancing Perovskite Electrocatalysis through Strain Tuning of the Oxygen Deficiency. J. Am. Chem. Soc. 2016, 138, 7252–7255. 10.1021/jacs.6b03520. 27232374

[ref12] Hansen B. B.; Spittle S.; Chen B.; Poe D.; Zhang Y.; Klein J. M.; Horton A.; Adhikari L.; Zelovich T.; Doherty B. W.; Gurkan B.; Maginn E. J.; Ragauskas A.; Dadmun M.; Zawodzinski T. A.; Baker G. A.; Tuckerman M. E.; Savinell R. F.; Sangoro J. R. Deep Eutectic Solvents : A Review of Fundamentals and Applications. Chem. Rev. 2021, 121, 1232–1285. 10.1021/acs.chemrev.0c00385. 33315380

[ref13] Abbott A. P.; Boothby D.; Capper G.; Davies D. L.; Rasheed R. K. Deep Eutectic Solvents Formed between Choline Chloride and Carboxylic Acids: Versatile Alternatives to Ionic Liquids. J. Am. Chem. Soc. 2004, 126, 9142–9147. 10.1021/ja048266j. 15264850

[ref14] Hong S.; Doughty R. M.; Osterloh F. E.; Zaikina J. V. Deep Eutectic Solvent Route Synthesis of Zinc and Copper Vanadate N-Type Semiconductors – Mapping Oxygen Vacancies and Their Effect on Photovoltage. J. Mater. Chem. A 2019, 7, 12303–12316. 10.1039/c9ta00957d.

[ref15] Hong S.; Burkhow S. J.; Doughty R. M.; Cheng Y.; Ryan B. J.; Mantravadi A.; Roling L. T.; Panthani M. G.; Osterloh F. E.; Smith E. A.; Zaikina J. V. Local Structural Disorder in Metavanadates MV_2_O_6_ (M = Zn and Cu): Photoactive Oxides with Oxygen Vacancies. Chem. Mater. 2021, 33, 1667–1682. 10.1021/acs.chemmater.0c04155.

[ref16] Man I. C.; Su H. Y.; Calle-Vallejo F.; Hansen H. A.; Martínez J. I.; Inoglu N. G.; Kitchin J.; Jaramillo T. F.; Nørskov J. K.; Rossmeisl J. Universality in Oxygen Evolution Electrocatalysis on Oxide Surfaces. ChemCatChem 2011, 3, 1159–1165. 10.1002/cctc.201000397.

[ref17] Suntivich J.; May K. J.; Gasteiger H. A.; Goodenough J. B.; Shao-Horn Y. A Perovskite Oxide Optimized for Oxygen Evolution Catalysis from Molecular Orbital Principles. Science 2011, 334, 1383 10.1126/science.1212858. 22033519

[ref18] Guo Y.; Zhang X.; Wäppling R. Crystal Structure of La_1–*x*_Sr_*x*_MnO_3–2*x*_+F_2*x*_. J. Alloys Compd. 2000, 306, 133–140. 10.1016/s0925-8388(00)00763-5.

[ref19] Haas O.; Struis R. P. W. J.; McBreen J. M. Synchrotron X-Ray Absorption of LaCoO_3_ Perovskite. J. Solid State Chem. 2004, 177, 1000–1010. 10.1016/j.jssc.2003.10.004.

[ref20] Megaw H. D.; Darlington C. N. W. Geometrical and Structural Relations in the Rhombohedral Perovskites. Acta Crystallogr. 1975, 31, 161–173. 10.1107/s0567739475000332.

[ref21] Blasco J.; Sánchez M. C.; Pérez-Cacho J.; García J.; Subías G.; Campo J. Synthesis and Structural Study of LaNi_1–*x*_Mn_*x*_O_3+δ_ Perovskites. J. Phys. Chem. Solids 2002, 63, 781–792. 10.1016/s0022-3697(01)00228-1.

[ref22] Bull C. L.; Gleeson D.; Knight K. S. Determination of B-Site Ordering and Structural Transformations in the Mixed Transition Metal Perovskites La_2_CoMnO_6_ and La_2_NiMnO_6_. J. Phys.: Condens. Matter 2003, 15, 4927 10.1088/0953-8984/15/29/304.

[ref23] Radaelli P. G.; Cheong S.-W. Structural Phenomena Associated with the Spin-State Transition in LaCoO_3_. Phys. Rev. B 2002, 66, 94408 10.1103/physrevb.66.094408.

[ref24] Alom M. S.; Ramezanipour F. Layered Oxides SrLaFe_1–*x*_Co_*x*_O_4−δ_ (X = 0–1) as Bifunctional Electrocatalysts for Water-Splitting. ChemCatChem 2021, 13, 3510–3516. 10.1002/cctc.202100867.

[ref25] Todd P. K.; Neilson J. R. Selective Formation of Yttrium Manganese Oxides through Kinetically Competent Assisted Metathesis Reactions. J. Am. Chem. Soc. 2019, 141, 1191–1195. 10.1021/jacs.8b10123. 30624059

[ref26] Todd P. K.; Wustrow A.; McAuliffe R. D.; McDermott M. J.; Tran G. T.; McBride B. C.; Boeding E. D.; O’Nolan D.; Liu C.-H.; Dwaraknath S. S.; Chapman K. W.; Billinge S. J. L.; Persson K. A.; Huq A.; Veith G. M.; Neilson J. R. Defect-Accommodating Intermediates Yield Selective Low-Temperature Synthesis of YMnO_3_ Polymorphs. Inorg. Chem. 2020, 59, 13639–13650. 10.1021/acs.inorgchem.0c02023. 32866379

[ref27] Jung S.; McCrory C. C. L.; Ferrer I. M.; Peters J. C.; Jaramillo T. F. Benchmarking nanoparticulate metal oxide electrocatalysts for the alkaline water oxidation reaction. J. Mater. Chem. A 2016, 4, 3068–3076. 10.1039/c5ta07586f.

[ref28] Macdonald J. R.; Johnson W. B. Fundamentals of Impedance Spectroscopy. In Impedance Spectroscopy; Barsoukov E., Macdonald J. R., Eds.; John Wiley & Sons, Ltd, 2018; pp 1–20.

[ref29] Bonanos N.; Steele B. C. H.; Butler E. P.; Macdonald J. R.; Johnson W. B.; Worrell W. L.; Niklasson G. A.; Malmgren S.; Strømme M.; Sundaram S. K.; McKubre M. C. H.; Macdonald D. D.; Engelhardt G. R.; Barsoukov E.; Conway B. E.; Pell W. G.; Wagner N.; Roland C. M.; Eisenberg R. S. Applications of Impedance Spectroscopy. In Impedance Spectroscopy; Barsoukov E., Macdonald J. R., Eds.; John Wiley & Sons, Ltd, 2018; p 175.

[ref30] Xia B.; Wang T.; Ran J.; Jiang S.; Gao X.; Gao D. Optimized Conductivity and Spin States in N-Doped LaCoO_3_ for Oxygen Electrocatalysis. ACS Appl. Mater. Interfaces 2021, 13, 2447–2454. 10.1021/acsami.0c16150. 33399444

[ref31] Aksenova T. V.; Efimova T. G.; Lebedev O. I.; Elkalashy S. I.; Urusova A. S.; Cherepanov V. A. Phase Equilibria, Crystal Structure and Properties of Complex Oxides in the Nd_2_O_3_–SrO–CoO System. J. Solid State Chem. 2017, 248, 183–191. 10.1016/j.jssc.2017.02.002.

[ref32] Jang J. H.; Kim Y.-M.; He Q.; Mishra R.; Qiao L.; Biegalski M. D.; Lupini A. R.; Pantelides S. T.; Pennycook S. J.; Kalinin S. V.; Borisevich A. Y. In Situ Observation of Oxygen Vacancy Dynamics and Ordering in the Epitaxial LaCoO3 System. ACS Nano 2017, 11, 6942–6949. 10.1021/acsnano.7b02188. 28602092

[ref33] Lebedev O. I.; Turner S.; Caignaert V.; Cherepanov V. A.; Raveau B. Exceptional Layered Ordering of Cobalt and Iron in Perovskites. Chem. Mater. 2016, 28, 2907–2911. 10.1021/acs.chemmater.6b01046.

[ref34] Zhang Q.; Gao A.; Meng F.; Jin Q.; Lin S.; Wang X.; Xiao D.; Wang C.; Jin K.-j.; Su D.; Guo E.-J.; Gu L. Near-Room Temperature Ferromagnetic Insulating State in Highly Distorted LaCoO_2.5_ with CoO_5_ Square Pyramids. Nat. Commun. 2021, 12, 1853 10.1038/s41467-021-22099-y. 33767171 PMC7994406

[ref35] Pfeifer V.; Jones T. E.; Velasco Vélez J. J.; Arrigo R.; Piccinin S.; Hävecker M.; Knop-Gericke A.; Schlögl R. In Situ Observation of Reactive Oxygen Species Forming on Oxygen-Evolving Iridium Surfaces. Chem. Sci. 2017, 8, 2143–2149. 10.1039/c6sc04622c. 28507666 PMC5407268

[ref36] Cheng X.; Fabbri E.; Nachtegaal M.; Castelli I. E.; El Kazzi M.; Haumont R.; Marzari N.; Schmidt T. J. Oxygen Evolution Reaction on La_1–*x*_Sr_*x*_CoO_3_ Perovskites: A Combined Experimental and Theoretical Study of Their Structural, Electronic, and Electrochemical Properties. Chem. Mater. 2015, 27, 7662–7672. 10.1021/acs.chemmater.5b03138.

[ref37] Binninger T.; Mohamed R.; Waltar K.; Fabbri E.; Levecque P.; Kötz R.; Schmidt T. J. Thermodynamic Explanation of the Universal Correlation between Oxygen Evolution Activity and Corrosion of Oxide Catalysts. Sci. Rep. 2015, 5, 12167 10.1038/srep12167. 26178185 PMC4503990

[ref38] Grimaud A.; Diaz-Morales O.; Han B.; Hong W. T.; Lee Y.-L.; Giordano L.; Stoerzinger K. A.; Koper M. T. M.; Shao-Horn Y. Activating Lattice Oxygen Redox Reactions in Metal Oxides to Catalyse Oxygen Evolution. Nat. Chem. 2017, 9, 457–465. 10.1038/nchem.2695. 28430191

[ref39] Lopes P. P.; Chung D. Y.; Rui X.; Zheng H.; He H.; Farinazzo Bergamo Dias Martins P.; Strmcnik D.; Stamenkovic V. R.; Zapol P.; Mitchell J. F.; Klie R. F.; Markovic N. M. Dynamically Stable Active Sites from Surface Evolution of Perovskite Materials during the Oxygen Evolution Reaction. J. Am. Chem. Soc. 2021, 143, 2741–2750. 10.1021/jacs.0c08959. 33399469

[ref40] Pan Y.; Xu X.; Zhong Y.; Ge L.; Chen Y.; Veder J.-P. M.; Guan D.; O’Hayre R.; Li M.; Wang G.; Wang H.; Zhou W.; Shao Z. Direct Evidence of Boosted Oxygen Evolution over Perovskite by Enhanced Lattice Oxygen Participation. Nat. Commun. 2020, 11, 2002 10.1038/s41467-020-15873-x. 32332731 PMC7181763

[ref41] Adler S. B. Factors Governing Oxygen Reduction in Solid Oxide Fuel Cell Cathodes. Chem. Rev. 2004, 104, 4791–4844. 10.1021/cr020724o. 15669169

[ref42] Pourbaix M. Atlas of Electrochemical Equilibria in Aqueous Solutions, 2nd English ed.; National Association of Corrosion Engineers: Houston, TX, 1974.

[ref43] Xu J.; Sousa J. P. S.; Mordvinova N. E.; Costa J. D.; Petrovykh D. Y.; Kovnir K.; Lebedev O. I.; Kolen’ko Y. V. Al-Induced In Situ Formation of Highly Active Nanostructured Water-Oxidation Electrocatalyst Based on Ni-Phosphide. ACS Catal. 2018, 8, 2595–2600. 10.1021/acscatal.7b03817.

[ref44] Xu J.; Wei X.-K.; Costa J. D.; Lado J. L.; Owens-Baird B.; Gonçalves L. P. L.; Fernandes S. P. S.; Heggen M.; Petrovykh D. Y.; Dunin-Borkowski R. E.; Kovnir K.; Kolen’ko Y. V. Interface Engineering in Nanostructured Nickel Phosphide Catalyst for Efficient and Stable Water Oxidation. ACS Catal. 2017, 7, 5450–5455. 10.1021/acscatal.7b01954.

[ref45] Rosser T. E.; Sousa J. P. S.; Ziouani Y.; Bondarchuk O.; Petrovykh D. Y.; Wei X.-K.; Humphrey J. J. L.; Heggen M.; Kolen’ko Y. V.; Wain A. J. Enhanced Oxygen Evolution Catalysis by Aluminium-Doped Cobalt Phosphide through in Situ Surface Area Increase. Catal. Sci. Technol. 2020, 10, 2398 10.1039/d0cy00123f.

[ref46] Mann D. K.; Xu J.; Mordvinova N. E.; Yannello V.; Ziouani Y.; González-Ballesteros N.; Sousa J. P. S.; Lebedev O. I.; Kolen’ko Y. V.; Shatruk M. Electrocatalytic Water Oxidation over AlFe_2_B_2_. Chem. Sci. 2019, 10, 2796–2804. 10.1039/c8sc04106g. 30997000 PMC6425857

[ref47] Zhu Y.; Lin Q.; Hu Z.; Chen Y.; Yin Y.; Tahini H. A.; Lin H. J.; Chen C. T.; Zhang X.; Shao Z.; Wang H. Self-Assembled Ruddlesden–Popper/Perovskite Hybrid with Lattice-Oxygen Activation as a Superior Oxygen Evolution Electrocatalyst. Small 2020, 16, 2001204 10.1002/smll.202001204. 32309914

[ref48] Baeumer C.; Li J.; Lu Q.; Liang A. Y.-L.; Jin L.; Martins H. P.; Duchoň T.; Glöß M.; Gericke S. M.; Wohlgemuth M. A.; Giesen M.; Penn E. E.; Dittmann R.; Gunkel F.; Waser R.; Bajdich M.; Nemšák S.; Mefford J. T.; Chueh W. C. Tuning Electrochemically Driven Surface Transformation in Atomically Flat LaNiO_3_ Thin Films for Enhanced Water Electrolysis. Nat. Mater. 2021, 20, 674–682. 10.1038/s41563-020-00877-1. 33432142

[ref49] Choi S. R.; Lee J.-I.; Park H.; Lee S. W.; Kim D. Y.; An W. Y.; Kim J. H.; Kim J.; Cho H.-S.; Park J.-Y. Multiple Perovskite Layered Lanthanum Nickelate Ruddlesden-Popper Systems as Highly Active Bifunctional Oxygen Catalysts. Chem. Eng. J. 2021, 409, 128226 10.1016/j.cej.2020.128226.

[ref50] Bhowmick S.; Dhankhar A.; Sahu T. K.; Jena R.; Gogoi D.; Peela N. R.; Ardo S.; Qureshi M. Low Overpotential and Stable Electrocatalytic Oxygen Evolution Reaction Utilizing Doped Perovskite Oxide, La_0.7_Sr_0.3_MnO_3_, Modified by Cobalt Phosphate. ACS Appl. Energy Mater. 2020, 3, 1279–1285. 10.1021/acsaem.9b02167.

[ref51] Dong F.; Li L.; Kong Z.; Xu X.; Zhang Y.; Gao Z.; Dongyang B.; Ni M.; Liu Q.; Lin Z. Materials Engineering in Perovskite for Optimized Oxygen Evolution Electrocatalysis in Alkaline Condition. Small 2021, 17, 2006638 10.1002/smll.202006638. 33325635

[ref52] Li T.; Guo W.; Shi Q. Development and Performance of A-Site Rich Perovskite-Type Material for Enhanced Oxygen Evolution Reaction in Alkaline Electrolyte. J. Mater. Sci.: Mater. Electron. 2020, 31, 21272–21278. 10.1007/s10854-020-04639-2.

[ref53] Li Z.; Xue K.-H.; Wang J.; Li J.-G.; Ao X.; Sun H.; Song X.; Lei W.; Cao Y.; Wang C. Cation and Anion Co-Doped Perovskite Nanofibers for Highly Efficient Electrocatalytic Oxygen Evolution. ACS Appl. Mater. Interfaces 2020, 12, 41259–41268. 10.1021/acsami.0c10045. 32841005

[ref54] Njoku C. B.; Doyle B. P.; Carleschi E.; Kriek R. J. Ce_0.8_Sr_0.2_Co_*x*_Fe_1–*x*_O_3−δ_ (*x*=0.2, 0.5, 0.8) – A Perovskite-Type Nanocomposite for Application in the Oxygen Evolution Reaction in Alkaline Media. Electroanalysis 2020, 32, 3131–3144. 10.1002/elan.202060370.

