# Supplementary material for: Deep Eutectic Solvent Synthesis of Perovskite Electrocatalysts for Water Oxidation
Source: ACS Appl Mater Interfaces. 2022 May 12;14(20):23277–84. doi: 10.1021/acsami.1c24223 (PMC9136838; doi:10.1021/acsami.1c24223)
Supplement: Supplementary file 1 — am1c24223_si_001.pdf [file am1c24223_si_001.pdf]

Supporting Information for

Deep Eutectic Solvent Synthesis of Perovskite Electrocatalysts for Water Oxidation

Sangki Hong,[|, #] *Aida M. Díez,*[⊥, #] *Adedoyin N. Adeyemi,*[|] *Juliana P. S. Sousa,*[⊥] *Laura M. Salonen,*[⊥]
*Oleg I. Lebedev,**[, ||] *Yury V. Kolen'ko,**[, ⊥] *and Julia V. Zaikina**[, |]

[|] Department of Chemistry, Iowa State University, Ames, Iowa 50011, United States
[⊥] Nanochemistry Research Group, International Iberian Nanotechnology Laboratory, Braga 4715-330, Portugal
[||] Laboratoire CRISMAT, UMR 6508, CNRS-ENSICAEN, Caen 14050, France
[#] equal contribution

oleg.lebedev@ensicaen.fr
yury.kolenko@inl.int
yzaikina@iastate.edu

EXPERIMENTAL

Materials

Malonic acid $CH_2(COOH)_2$ (Alfa Aesar, 99.5%), $LaCl_3$ (Alfa Aesar, 99.9%), $CoCl_2$ (Alfa Aesar, 97%), $NiCl_2$ (Alfa Aesar, 98%), Mn_2O_3 (Sigma-Aldrich, 99.9%) were used as received without any further purification. Choline chloride (Sigma-Aldrich, ≥98%) was dried at 120 °C under vacuum prior to use.

Characterization

Powder X-ray Diffraction

Samples were characterized by powder X-ray diffraction (PXRD) using a Miniflex 600 diffractometer (Rigaku) with Cu K_α radiation (λ = 1.54051 Å). Diffraction scan was collected from 5 to 90° 2θ on a zero-background plate at room temperature in air. Phase identification was performed using the PDF-2 database using PDXL[1] and Match[2] software packages. The Rietveld refinement was carried out using the GSAS[3] and Jana 2006[4] software packages.

Electron Microscopy

The transmission electron microscopy (TEM), high-resolution TEM (HRTEM), high-angle annular dark-field scanning TEM (HAADF–STEM), selected area electron diffraction (SAED) and energy-dispersive X-ray spectroscopy in STEM mode (STEM–EDX) investigations were performed using a JEM-ARM200F microscope (Jeol) operated at 200 kV and equipped with cold field-emission gun, probe and image aberration correction, a CENTURIO EDX detector, and a GIF Quantum filter.

Chemical Analysis

The inductively coupled plasma–optical emission spectroscopy (ICP–OES) was carried out using the ICPE-9000 spectrometer (Shimadzu). Each sample was measured three times to ensure reproducibility of results.

Electrocatalytic Testing

Anode Preparation

To facilitate electrocatalytic ink formulation, DES-derived perovskites were first ball-milled at 1725 rpm for 1 h in an 8000 M High-Energy Mixer/Mill (SPEX), using a 5 mL tungsten carbide lined grinding vial set. The ball-milled samples were then formulated into an electrocatalyst ink by dispersing 5 mg of the material in 50 µL of Nafion ionomer solution (5% in aliphatic alcohols and water, Sigma-Aldrich) and 1.0 mL of anhydrous ethanol (Honeywell). The mixture was first

homogenized in a bath sonicator USC-TH (VWR) for 30 min, and then using an ultrasonic probe Vibra-cell 75185 (Thermo Fisher Scientific) for 1 min.

Electrocatalytic anodes containing perovskite materials and the reference IrO_2 electrocatalyst (99.99%, Alfa Aesar) were prepared by loading the ink on a Ni foam supporting material (Heze Jiaotong, 110 pores per inch, 0.3 mm thick). Prior ink deposition, Ni foam was cleaned by sequential 30-min ultrasonication in acetone, ethanol, and Milli-Q water. The ink was loaded in 20 μL batches on the surface of the Ni foam current collector, while letting ethanol to evaporate between the batches. The exposed geometrical surface area of the anode was fixed to be a 1 cm^2 and the total mass of electrocatalyst loaded on the anode was varied from 0.25 to 3 mg cm^{-2}. Finally, the obtained anode was air-dried and subjected to electrochemical testing.

Electrochemical Measurements

Electrochemical studies were conducted at room temperature using Autolab PGSTAT302N potentiostat/galvanostat (Methrohm), equipped with a FRA32M frequency response analyzer. The performance of the electrocatalysts in oxygen evolution reaction (OER) was evaluated under moderate Ar bubbling (1 bubble s^{-1}) while stirring at 150 rpm in a three-electrode system filled with purified 1 M NaOH aqueous electrolyte solution. The electrocatalytic anode, a calibrated saturated calomel electrode (SCE), and a Pt wire served as the working, reference, and counter electrodes, respectively. All potentials reported in the work were converted to a reversible hydrogen electrode (RHE) reference scale according to the following equation: $E_{RHE} = E_{SCE} + 0.059pH + 0.241$. An iR-correction of 85% was applied in the polarization experiments to compensate for the voltage drop between the reference and working electrodes, which was estimated by a single-point high-frequency impedance measurement.

OER anodic polarization curves were recorded using cyclic voltammetry (CV) with a scan rate of 5 mV s^{-1}. In the case of electrocatalyst activation, the scan rate was augmented to 50 mV s^{-1}. Electrochemical impedance spectroscopy (EIS) measurements were carried out at the overpotential (η_{10}) that provide the current density of 10 mA cm^{-2} in the frequency range from 105 to 0.01 Hz with a 10 mV sinusoidal perturbation. The EIS measurements and the interpretation of results were conducted in accordance with aqueous electrochemical assembly, the so-called supported system. The electrocatalytic stability of the anodes was evaluated as a function of time by means of chronopotentiometry at constant current density of 10 mA cm^{-2}.

The relative electrochemically active surface area (ECSA) of the solid–electrolyte interface of the anodes was assessed on the basis of geometric double-layer capacitance, C_{dl}, measurements. CV

cycles were recorded from 1.17 V_{RHE} to 1.27 V_{RHE} for all perovskite electrodes under the standard conditions of alkaline OER experiment using increasing scan rates of 5, 10, 25, 50 and 100 mV s^{-1}. A linear trend is obtained via plotting half the difference in current density, j, between the anodic and cathodic sweeps, ½ ($j_{anodic} - j_{cathodic}$), in the non-Faradaic region of the cyclic voltammograms (ca. 1.2 V) as a function of the scan rate. The slope of the linear fitting of these data is the geometric C_{dl} (mF cm^{-2}), which is linearly proportional to the ECSA for a given surface.

TABLES

Table S1. Structural parameters of the DES-derived perovskites determined by Rietveld refinement using powder X-ray diffraction data.

Parameter	$LaMnO_3$	$LaCoO_3$	$LaMn_{0.5}Ni_{0.5}O_3$
**Space group**	$R\overline{3}c$		$P2_1/c$ *
a **(Å)**	5.5148(6)	5.4423(4)	5.462(8)
b **(Å)**	5.5148(6)	5.4423(4)	5.5110(9)
c **(Å)**	13.3425(7)	13.0980(6)	9.484(21)
**β (°)**			124.985(20)
**Volume (Å^3)**	351.42(5)	335.97(4)	233.90(10)
**G.O.F.**	1.10	1.15	1.01
R_w, %	6.50	5.86	5.54
R_p, %	5.10	4.55	4.23

* Structure reported by Blasco, *et. al.*[5] and Bull, *et. al.*[6] is in $P2_1/n$ space group, which is non-standard settings for $P2_1/c$ space group used here.

Table S2. Crystallite sizes of the as-synthesized and ball-milled perovskites, as estimated using the Scherrer equation ($K = 0.94$) and full width at half maximum (FWHM) of the diffraction peaks between $2\theta = 20°–60°$ (peak fitting carried out using GSAS[3] and Jana 2006[4] software packages).

**Samples**	**Crystallite size (nm)**	
	**before ball-milling**	**after ball-milling**
**$LaCoO_3$**	50(4)	46(7)
**$LaMnO_3$**	38(8)	36(5)
**$LaNi_{0.5}Mn_{0.5}O_3$**	30(8)	28(8)

Table S3. Electrocatalytic alkaline OER properties of perovskites synthesized by DES synthesis route followed by ball-milling.

**Electrocatalyst**	**$LaMnO_3$**	**$LaMn_{0.5}Ni_{0.5}O_3$**	**$LaCoO_3$**	**Reference IrO_2**
**Mass loading [a] [mg cm^{-1}]**	0.25	0.25	0.25	0.25
**Required number of activation cycles [b]**	700			0
**Potential of pre-oxidation peak [V]**	1.48 (3.13 mA)	1.48 (3.27 mA)	1.51 (5.2 mA)	–
**Overpotential η_{10} at current density of 10 mA cm^{-2} [V]**	0.40	0.40	0.39	0.42
**Overpotential η_{50} at current density of 50 mA cm^{-2} [V]**	0.46	0.46	0.43	0.50
**Overpotential η_{100} at current density of 100 mA cm^{-2} [V]**	–	–	0.47	0.54
**Tafel slope b [c] [mV dec^{-1}]**	65.8	60.3	55.8	70.4
**Equivalent series resistance R_s [d] [Ω]**	0.8548	0.8163	1.0915	0.985
**Electron/charge transport resistance R_1 [d] [Ω]**	0.1784	0.1119	0.1004	1.860
**Charge transfer resistance R_{ct} [d] [Ω]**	2.7260	2.6762	2.1692	2.820
**Double-layer capacitance C_{dl} [e] [mF cm^{-2}]**	5.8	4.1	7.7	–

[a] Optimal mass loading of perovskites was estimated experimentally measuring alkaline OER activity as a function of mass loading of the electrocatalyst (Figure S5).

[b] Estimated experimentally using CV cycling with scan rate of 50 mV s^{-1} in potential range of 1.06–1.80 V (Figure S6).

[c] Estimated by Tafel fit (Figure 2b) of anodic polarization curves (Figure 2a) of the respective electrocatalysts.

[d] Estimated by fitting the Nyquist plots (Figure 2c), measured at $\approx\eta_{10}$, to the equivalent circuit model (Figure 2c, inset).

[e] Estimated by linear fitting of the data representing anodic and cathodic difference in current density as a function of cyclic voltammetry scan rate (Figure S7).

FIGURES

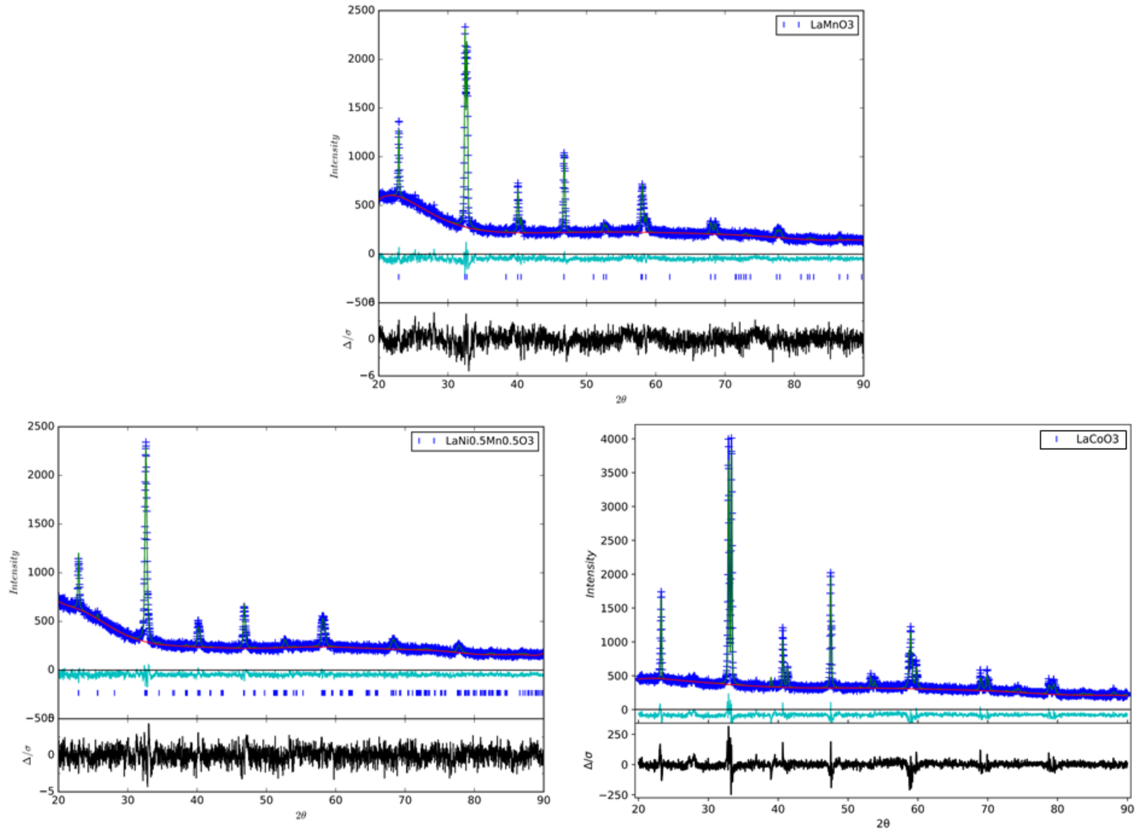

Figure S1. Rietveld refinement plots of powder X-ray diffraction patterns of DES-derived perovskites collected at room temperature; experimental powder patterns are in blue, calculated patterns are in green, differences are in black.

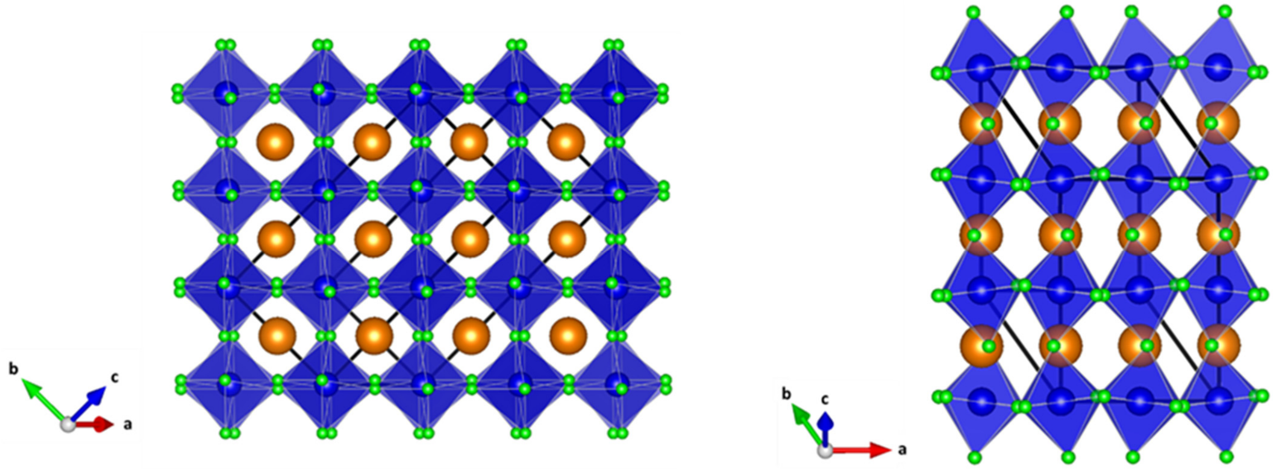

Figure S2. Rhombohedral crystal structure of LaTO$_3$ (T = Mn or Co). La: orange; Mn or Co: blue; O: green.

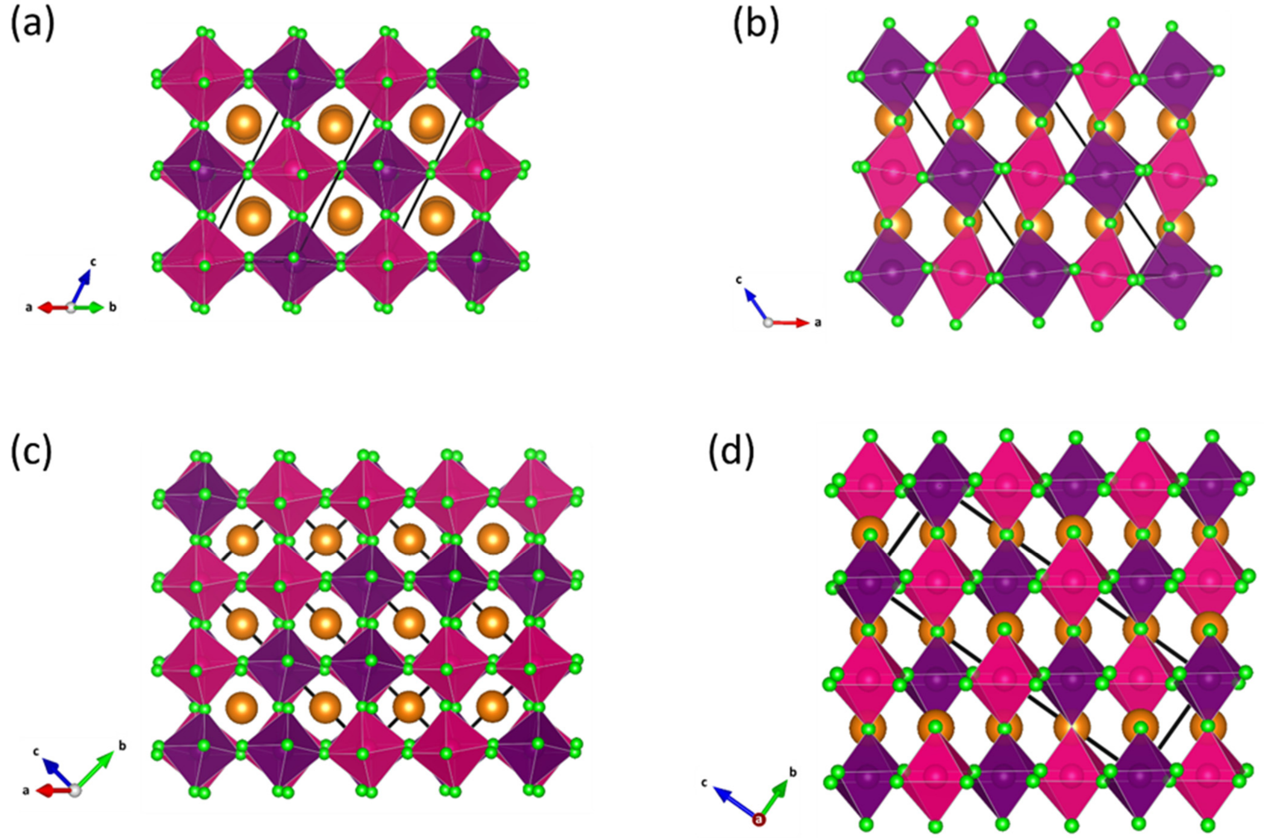

Figure S3. Crystal structure of double perovskite $LaMn_{0.5}Ni_{0.5}O_3$ (nominal formula: La_2MnNiO_6): monoclinic phase $P2_1/c$ (a,b) and rhombohedral phase $R\overline{3}$ (c,d). La: orange, Mn: purple, Ni: pink, O: green.

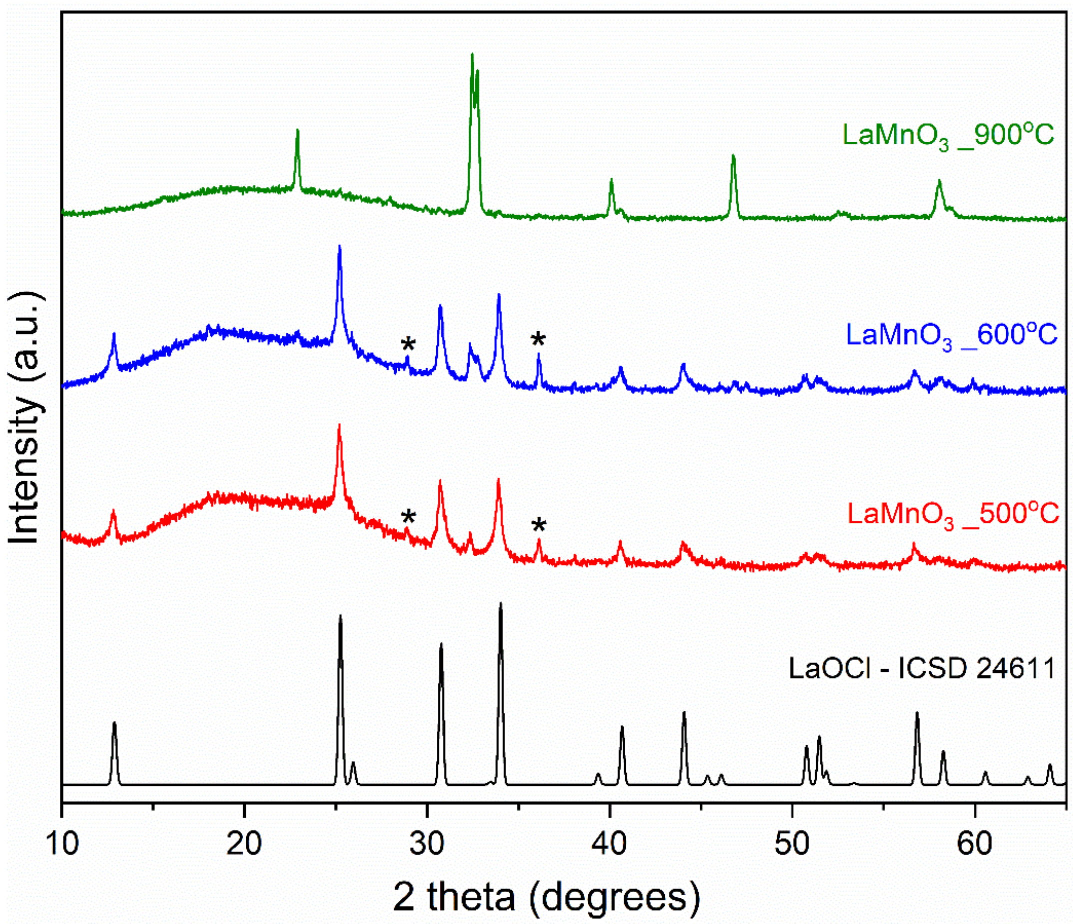

Figure S4. Powder X-ray diffraction patterns confirming the LaOCl intermediate formation during the synthesis of $LaMnO_3$ at different temperatures. The peaks of Mn_3O_4 are marked with asterisk (*).

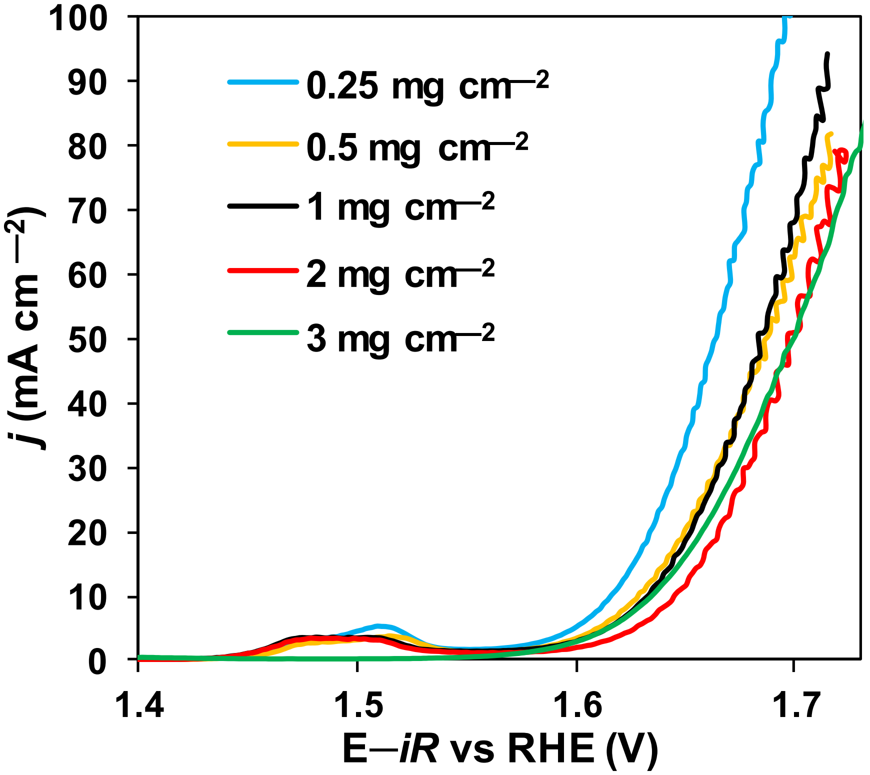

Figure S5. Alkaline OER activity of Ni-foam-supported $LaCoO_3$ as a function of the material mass loading. Anodic polarization curves are recorded after activation for 700 CV cycles.

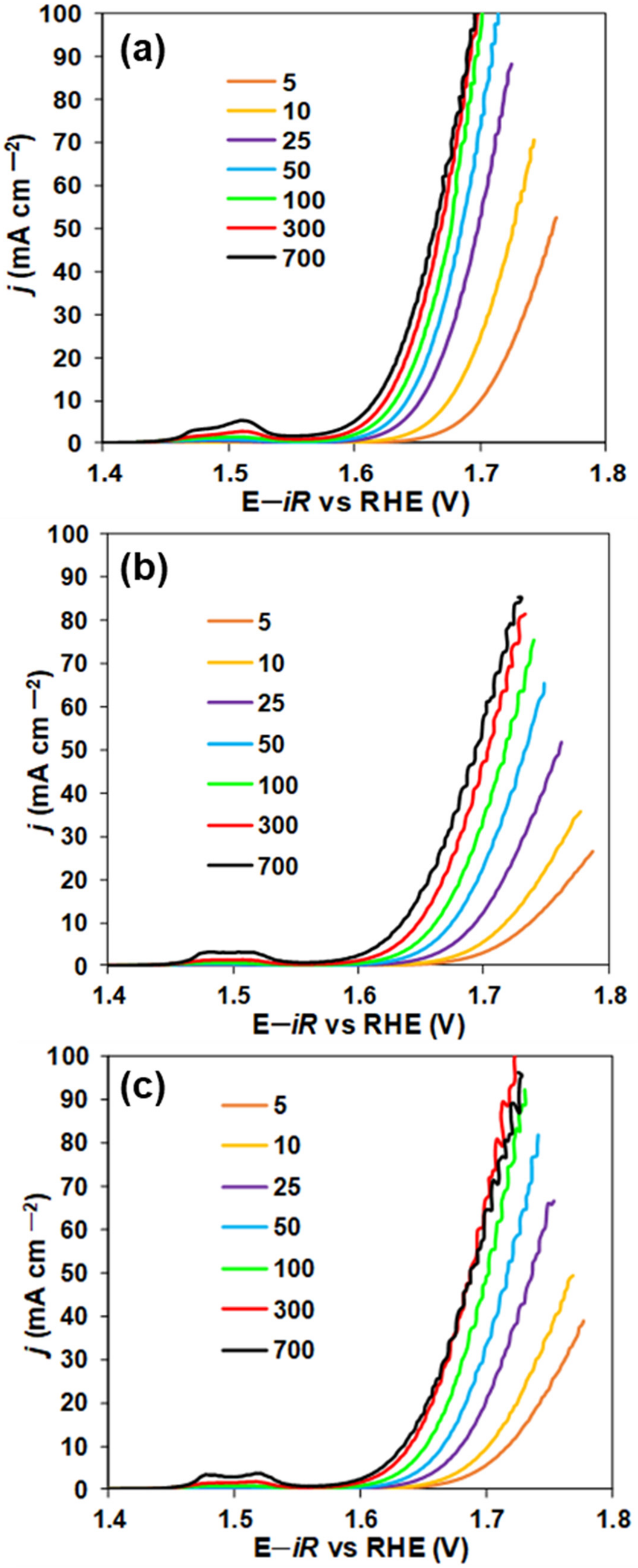

Figure S6. The current density vs. applied potential curves recorded over the Ni-foam-supported $LaCoO_3$ (a), $LaMnO_3$ (b) and $LaNi_{0.5}Mn_{0.5}O_3$ (c) electrocatalyst after specified numbers of alkaline OER activation CV cycles.

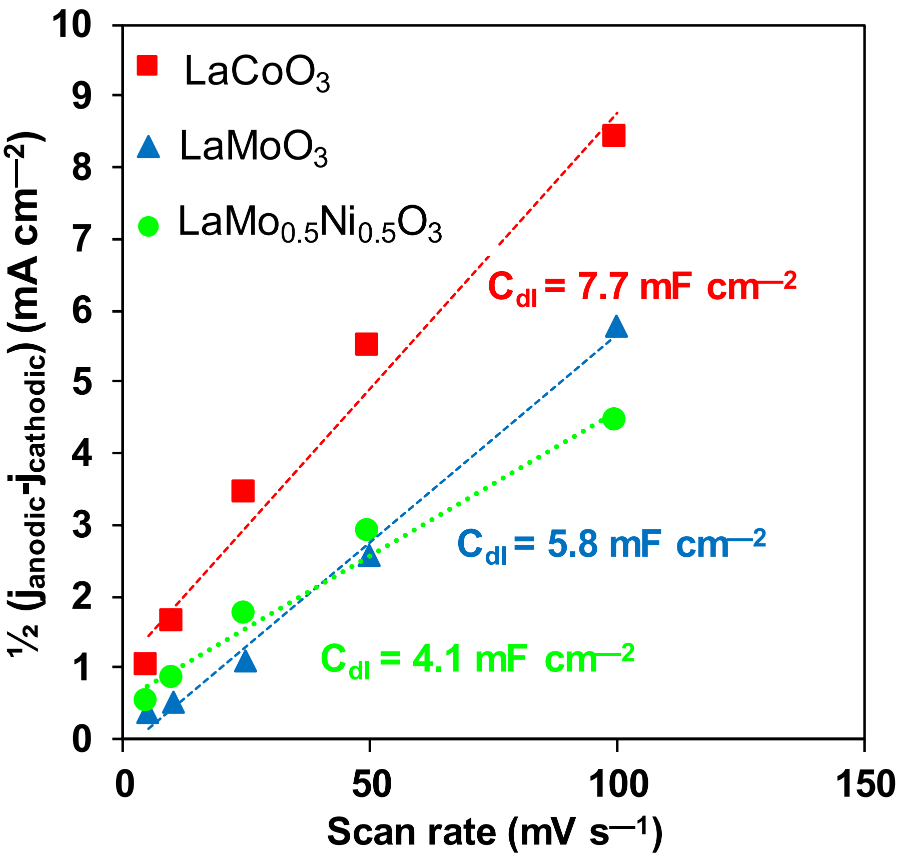

Figure S7. Geometric double-layer capacitance plots for the synthesized perovskite electrocatalysts. Raw data of cyclic voltammetry scans vs. scan rates were recorded in 1 M NaOH at room temperature with mass loading of 0.25 mg cm^{-2} after electrocatalyst activation for 700 CV cycles.

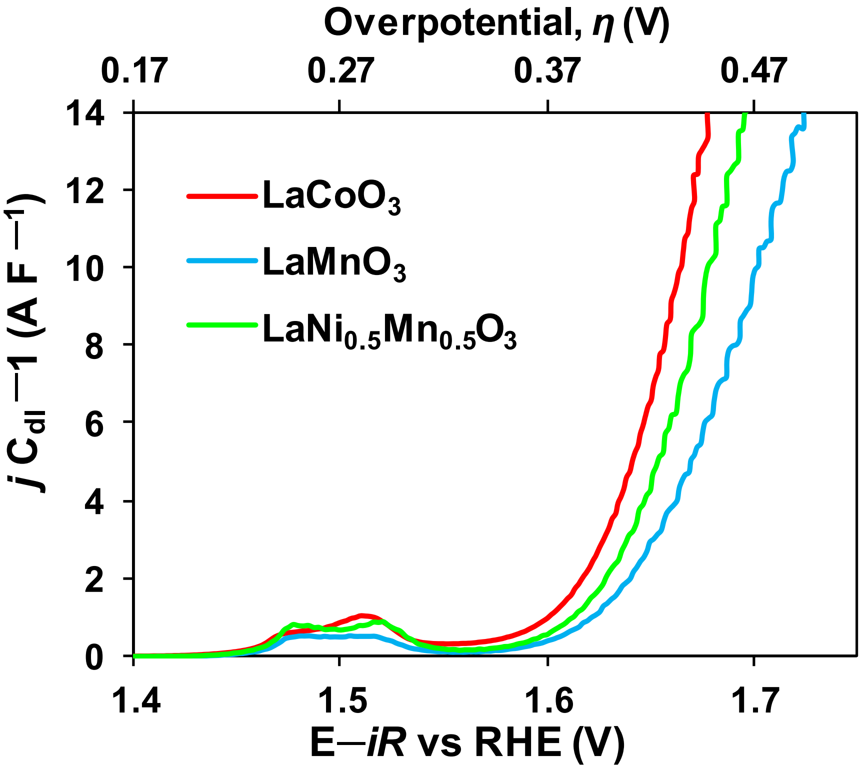

Figure S8. Anodic polarization OER data of DES-derived perovskites presented in Figure 2a, with currents normalized to double layer capacitance calculated in Figure S7.

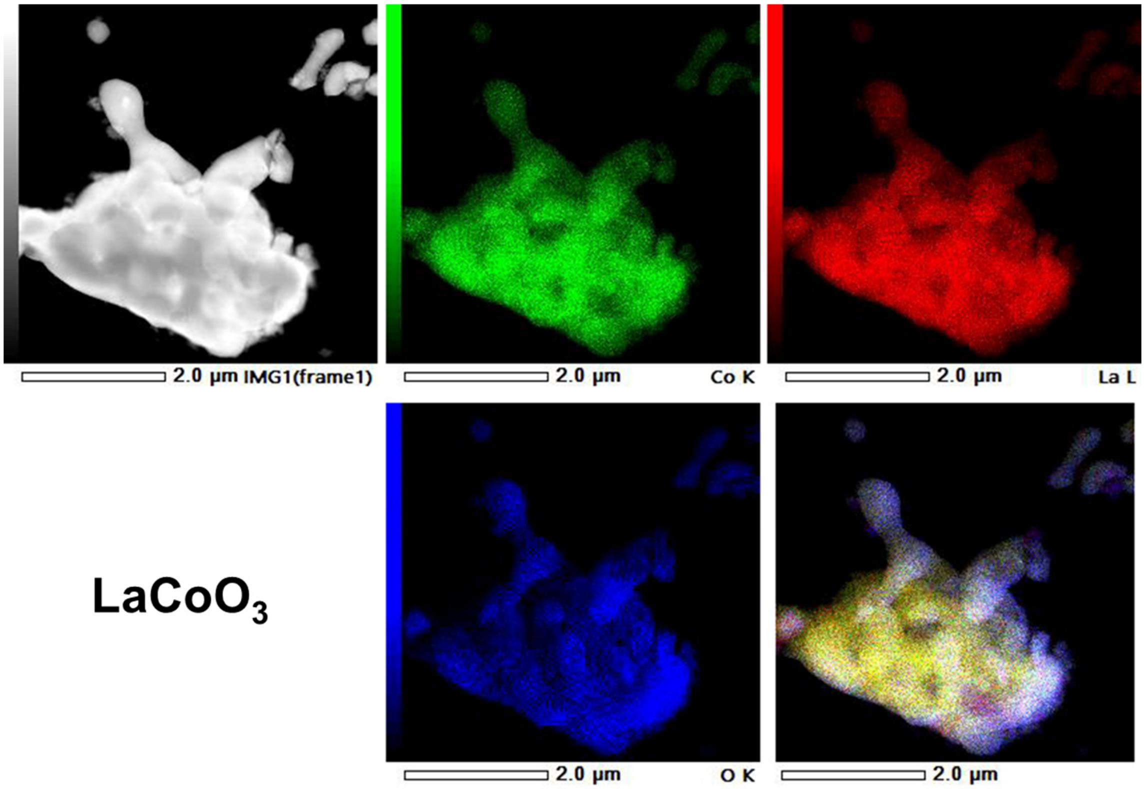

Figure S9. HAADF–STEM image of $LaCoO_3$ prepared by the DES synthesis route followed by ball-milling, together with the simultaneously collected STEM–EDX elemental maps of Co, La, O, and their mixture, indicating quite a homogeneous distribution of all the elements within the sample.

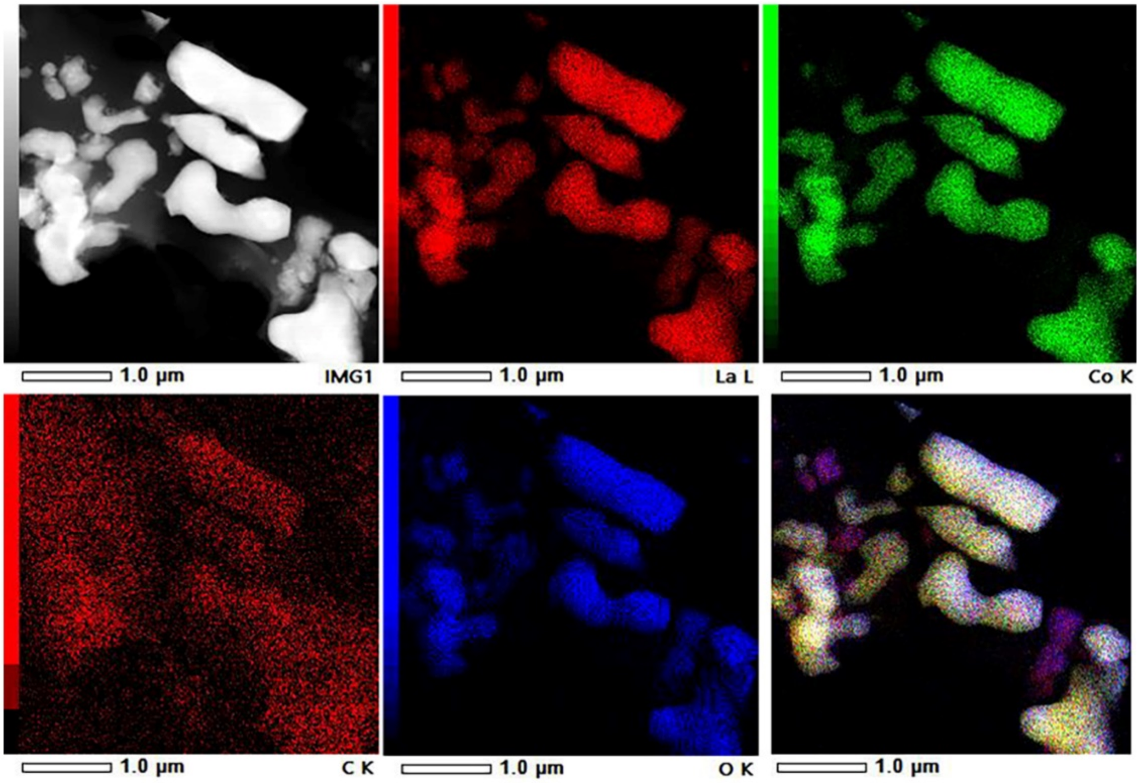

Figure S10. HAADF–STEM image of $LaCoO_3$ after stability testing in alkaline OER for 100 h (Figure 2d), together with the simultaneously collected STEM–EDX mappings of La, Co, C, O, and their mixture. The appearance of C stems from conductive Nafion ionomer, which was used for the anchoring of $LaCoO_3$ to Ni foam current collector. Note that the mixture of the STEM–EDX mappings reveals the segregation of La- and O-containing phase [most likely $La(OH)_3$] after OER stability testing, seen as violet regions in the mixture (bottom right).

REFERENCES

1. PDXL: Integrated X-ray powder diffraction software, Version 2.8.1.1. Rigaku, 2018.
2. H. Putz, K. Brandenburg, Match! -Phase analysis using powder diffraction, Crystal impact.
3. B. H. Toby, R. B. Von Dreele, GSAS-II: the genesis of a modern open-source all purpose crystallography software package. *J. Appl. Cryst*. 2013, **46**, 544-549.
4. V. Petricek, M. Dusek, L. Palatinus, Crystallographic computing system Jana2006: General features. Z. Kristallogr. 2014, **229**, 345-352.
5. J. Blasco, M.C. Sánchez, J. Pérez-Cacho, J. García, G. Subías, J. Campo. Synthesis and Structural Study of $LaNi_{1-x}Mn_xO_{3+\delta}$ Perovskites. *J. Phys. Chem. Solids* **2002**, *63*, 781–792.
6. C. L. Bull, D. Gleeson, K.S. Knight. Determination of B-Site Ordering and Structural Transformations in the Mixed Transition Metal Perovskites La_2CoMnO_6 and La_2NiMnO_6. *J. Phys. Condens. Matter* **2003**, *15*, 4927.
